# Exploring the role of hemiarthroplasty in revision shoulder arthroplasty: a systematic review

**DOI:** 10.1186/s10195-025-00883-6

**Published:** 2025-09-26

**Authors:** Gabrieleanselmo Uccheddu, Marco Verona, Filip Dąbrowski, Tomasz Mazurek, Antonio Capone, Giuseppe Marongiu

**Affiliations:** 1 Orthopaedic and Trauma Clinic, Department of Surgical Sciences, Policlinico Duilio Casula, Cagliari State University, 09042 Cagliari, Italy; 2 Department of Orthopaedics and Traumatology, Faculty of Medicine, Medical University of Gdansk, 80-803 Gdansk, Poland

**Keywords:** Hemiarthroplasty, Revision shoulder arthroplasty, Glenoid loosening, Reverse total shoulder arthroplasty, Anatomic shoulder arthroplasty, Shoulder salvage surgery, Functional outcomes

## Abstract

**Background:**

Hemiarthroplasty (HA) is a salvage option in revision shoulder arthroplasty when reimplantation (aTSA/rTSA) or secure glenoid fixation is not feasible. This systematic review evaluates indications, clinical outcomes, and complications after conversion to HA using an indication- and implant-stratified synthesis.

**Methods:**

Following Preferred Reporting Items for Systematic Reviews and Meta-Analyses (PRISMA), PubMed/MEDLINE, Embase/Scopus, and Web of Science were queried to 15 March 2024. Studies reporting revision of any shoulder arthroplasty to HA with ≥ 12-month follow-up were included. Owing to heterogeneity in measures and implant types, a descriptive analysis stratified by initial implant × indication was performed; primary endpoints were postoperative functional scores, with complications and reoperations as secondary endpoints.

**Results:**

Of 580 identified studies, 20 met inclusion criteria, totaling 268 patients. Glenoid component loosening was the most frequent indication (≈59%), followed by soft-tissue insufficiency (≈11%) and infection (≈9%). Postoperative function varied: ASES 48.2–66, constant 22–37, SANE 54–70. Complications occurred in 29%, and 15.7% underwent reoperation. Outcomes were indication-dependent: the highest scores were observed after humeral loosening (small subgroup), whereas glenoid loosening after aTSA or rTSA showed moderate, clinically meaningful improvements, particularly when bone loss could be reconstructed (e.g., grafting). Instability yielded modest gains, and infection was associated with the poorest results. Preoperative values were inconsistently reported, limiting Δ estimates.

**Conclusions:**

HA remains a salvage solution with indication-dependent effectiveness: best after humeral/glenoid loosening when reconstruction is feasible, modest in instability, and poor in infection. While HA can relieve pain and provide moderate functional improvement, it does not restore normal function. Selection should be deliberate and indication-specific, and future studies should adopt standardized reporting and prospective, indication-stratified designs.

## Introduction

Shoulder arthroplasty has become a widely accepted surgical intervention for various shoulder pathologies, offering significant pain relief and functional improvement for patients [1–3]. The prevalence of primary implants has increased rapidly in recent years [4–6] accompanied by a significant shift in the frequency of the most commonly used prosthesis [4, 5] resulting in a widespread use of reverse total shoulder arthroplasty (rTSA) for the treatment of fractures in elders [7–11], fracture sequelae [12, 13] and cuff tear arthropathy. Anatomic total shoulder arthroplasty (aTSA) is primarily indicated for the management of primary osteoarthritis [14] and selected cases of fracture sequelae [12, 13]. In contrast, hemiarthroplasty (HA), which involves the replacement of only the humeral head, is often the preferred treatment in young patients presenting with an irreparable articular fracture of the proximal humeral epiphysis [15, 16], avascular necrosis of the humeral head [1, 15] or osteoarthritis in which the glenoid remains unaffected [15].

The change in the prevalence of primary arthroplasties has been accompanied by a corresponding shift in revision rates [17], primarily owing to complications or failures of the previous implants [18–20].

When performing revision shoulder surgery, if the revision ends with the implantation of a rTSA, the results appear to be more reliable due to the fixation provided by the metal baseplate which also allows to address the problem of glenoid bone deficiency [19–22], while the semi-constrained design helps mitigate soft tissue imbalance and instability [20, 23].

In cases of substantial glenoid bone loss, often associated with failure of previous implants [24, 25], recurrent instability of aTSA, and rTSA, or infections, and when the patient’s conditions suggests a less invasive approach, several options can be considered: hemiarthroplasty [19, 26–28], resection arthroplasty (RA), arthrodesis, and retention of antibiotic spacer.

Hemiarthroplasty has been shown to be more reliable in terms of range of motion (ROM) and pain control compared with resection arthroplasty and shoulder arthrodesis [29]. Furthermore in those cases wherein the problem surges from the glenoid implant, a well-fixed humeral stem can be retained and converted into a hemiarthroplasty, allowing practitioners to perform a less invasive and challenging surgery than a revision to rTSA [30].

The latest designs of HA include the bipolar and the extended humeral head prostheses. Compared to traditional hemiarthroplasties, these implants provide a larger contact area with the glenoid and the coracoacromial (CA) arch, resulting in a decreased risk of antero-superior escape [31].

The following article is a systematic review and critical analysis of the literature regarding the revision of shoulder arthroplasty to HA and delves into the role of HA in revision shoulder surgery, its indications, and outcomes.

## Materials and methods

A systematic review was conducted following the Preferred Reporting Items for Systematic Reviews and MetaAnalyses (PRISMA) guidelines [32]. The articles were selected from PubMed/MEDLINE, Embase/Scopus, and Web of Science/Clarivate medical databases in March 2024. The search terms used for each database were the following: (((shoulder) AND (HA)) AND (revision)) AND (surgery), ((shoulder) AND (HA)) AND (salvage) from the latest to the most recent study published in PubMed until 15 March 2024; KEY (shoulder) AND KEY (HA) AND KEY (revision) AND KEY (surgery) AND PUBYEAR < 2025, KEY (shoulder) AND KEY (HA) AND KEY (salvage) AND PUBYEAR < 2025 from the latest to the most recent study published in Scopus until 15 March 2024; shoulder (Author Keywords) and revision (All Fields) and HA (All Fields) and surgery (All Fields), shoulder (Author Keywords) and HA (All Fields) and salvage (All Fields) from the latest to the most recent study published in Web of Science until 15 March 2024.

All Level I–IV studies in English language, which were published between 1988 and 15 March 2024, were considered for inclusion.

Inclusion criteria were conversion as a salvage or revision procedure of any kind of shoulder arthroplasty to HA for every reported indication with a minimum 12 months follow-up.

Exclusion criteria were the following: conversion or revision to any arthroplasty other than shoulder HA, less than 12 months radiological or clinical follow-up; use of national registries, reviews, editorials, technique articles, and case reports were excluded, articles published in languages other than English were excluded.

A total of 580 articles were selected from the above-mentioned databases, the removal of duplicates was performed with the aid of Rayyan free software, and 489 articles were left for revision.

All pertinent articles were meticulously cross-referenced manually by two independent reviewers (GU and GM) to guarantee comprehensive inclusion. Studies encompassing patient cohorts that met our inclusion criteria alongside those that did not were still incorporated; however, only data concerning patients who met our specific criteria were considered. To prevent redundancy, patient data appearing in multiple studies were included only once—extracted data comprised demographic details and clinical outcomes. Demographic data encompassed the total case count as well as the minimum and average follow-up durations, eventual reinterventions were reported including the number of cases if the original paper mentioned the data in question.

Indications to conversion/revision were regrouped into nine categories according to the main paper data, some authors did not expand on the main indication for intervention, therefore those patients that had a combination of two or more diagnoses were included in two or more categories in Tables 1 and 2.

**Table 1:** Study characteristics and cohorts

I author	Type of conversion	Indication	No. cases	Functional scale	M f-up	Complications	Reoperation
Seitz	aTSA → spacer → Hemi	Infection	5	University of Pennsylvania score	4.8 y	NR	NR
Sajadi	aTSA → Hemi; Hemi → Hemi	Glenoid loosening; humeral loosening; instability; soft-tissue failure	7 2 2 4	UCLA	27.6 m	2	2
Melis	aTSA → rTSA → Hemi	Instability	1	NR	47 m	NR	NR
Gamradt	rTSA → Hemi	Instability + infection; glenoid loosening	1 3	ASES; SST	26.5 m	4	NR
Boileau	rTSA → Hemi	Instability; Glenoid loosening	1 1	NR	36 m	NR	NR
Chalmers	rTSA → Hemi	Instability	2	NR	2.5 y	NR	NR
Al-Hadithy	rTSA → Hemi	Glenoid loosening	1	Constant; Oxford	5 y	NR	NR
Glanzmann	rTSA → Hemi	Infection; Glenoid loosening; periprosthetic humeral fracture; periprosthetic scapular fracture	3 8 2 4	Constant; SPADI; Quick DASH	2 y	3	1
Streubel	HHR → Hemi	Glenoid cartilage consumption	1	Modified Neer Score	3.5 y	NR	NR
Aibinder	aTSA → Hemi	Glenoid loosening	11	Modified Neer Score	8.3 y	4	2
Grubhofer	Hemi, rTSA, aTSA → spacer → Hemi	Infection	8	Constant	52 m	10	5
Lädermann	rTSA → Hemi	Glenoid loosening	28	Constant	2 y	18	NR
Song	rTSA → Hemi	Instability; Glenoid loosening	3 3	ASES; UCLA	24 m	NR	NR
Namdari	aTSA → Hemi	Glenoid loosening	17	ASES; SANE	40 m	6	5
Gauci	Hemi → Hemi; aTSA → Hemi; rTSA → Hemi	Stiffness; Soft tissue failure; Glenoid loosening; Infection Glenoid loosening	19 20 48 4 8	NR	8.7 y	20	20
Kriechling	rTSA → Hemi	Glenoid loosening; Instability; periprosthetic scapular fracture	8 2 2	Constant; SSV	46 m	2	NR
Guarrella	rTSA → Hemi	Instability	2	Constant	8 y	NR	NR
Khoo	aTSA → Hemi	Soft tissue failure + humeral loosening; soft tissue failure + glenoid loosening;glenoid loosening + humeral loosening; Glenoid loosening;	3 3 5 18	SST; ASES; SANE	12 m	7	7
Nezwek	aTSA → Hemi; rTSA → Hemi	Glenoid loosening Instability + glenoid loosening; infection;	1 3 3	SST; ASES; SANE/SSV	12 m	1	NR
John	rTSA → Hemi	Glenoid loosening	2	ASES; SST	31 m	NR	NR
Tot			268			77	37

*aTSA* anatomic total shoulder arthroplasty; *rTSA* reverse total shoulder arthroplasty; *Hemi* = *HA* *HHR*  humeral head resurfacing; *NR* not reported; *UCLA* University of California Los Angeles; *ASES* American Shoulder and Elbow Surgeons; *SST* Simple Shoulder Test; *SSV* subjective shoulder value; *SPADI* shoulder pain and disability index; *DASH* disability of the arm, shoulder, and hand

**Table 2:** Indications stratified by implant

Indication to conversion/revision	No. cases (%)
*aTSA → Hemi*	124 (46)
Glenoid loosening	110 (89)
Humeral loosening	10 (08)
Soft tissue failure	6 (05)
Infection	9 (07)
*rTSA → Hemi*	90 (34)
Glenoid loosening	65 (62)
Periprostetic scapular fracture	6 (06)
Periprostetic humeral fracture	2 (02)
Infection	7 (07)
Instability	15 (17)
*Hemi → Hemi*	45 (17)
Instability	2 (04)
Soft tissue failure	24 (53)
Stiffness	19 (42)
*HHR → Hemi*	1 (00)
Glenoid cartilage consumption	1 (100)
*Not specified → Hemi*	8 (03)
Infection	8 (100)

Data regarding the final implant and eventual biological resurfacing performed on the glenoid were collected and described in Fig. 2.

## Results

The search produced 580 articles from PubMed/MEDLINE, Embase/Scopus, and Web of Science/Clarivate medical databases; after duplicates had been removed, 489 articles were left for revision.

**Fig. 1:**
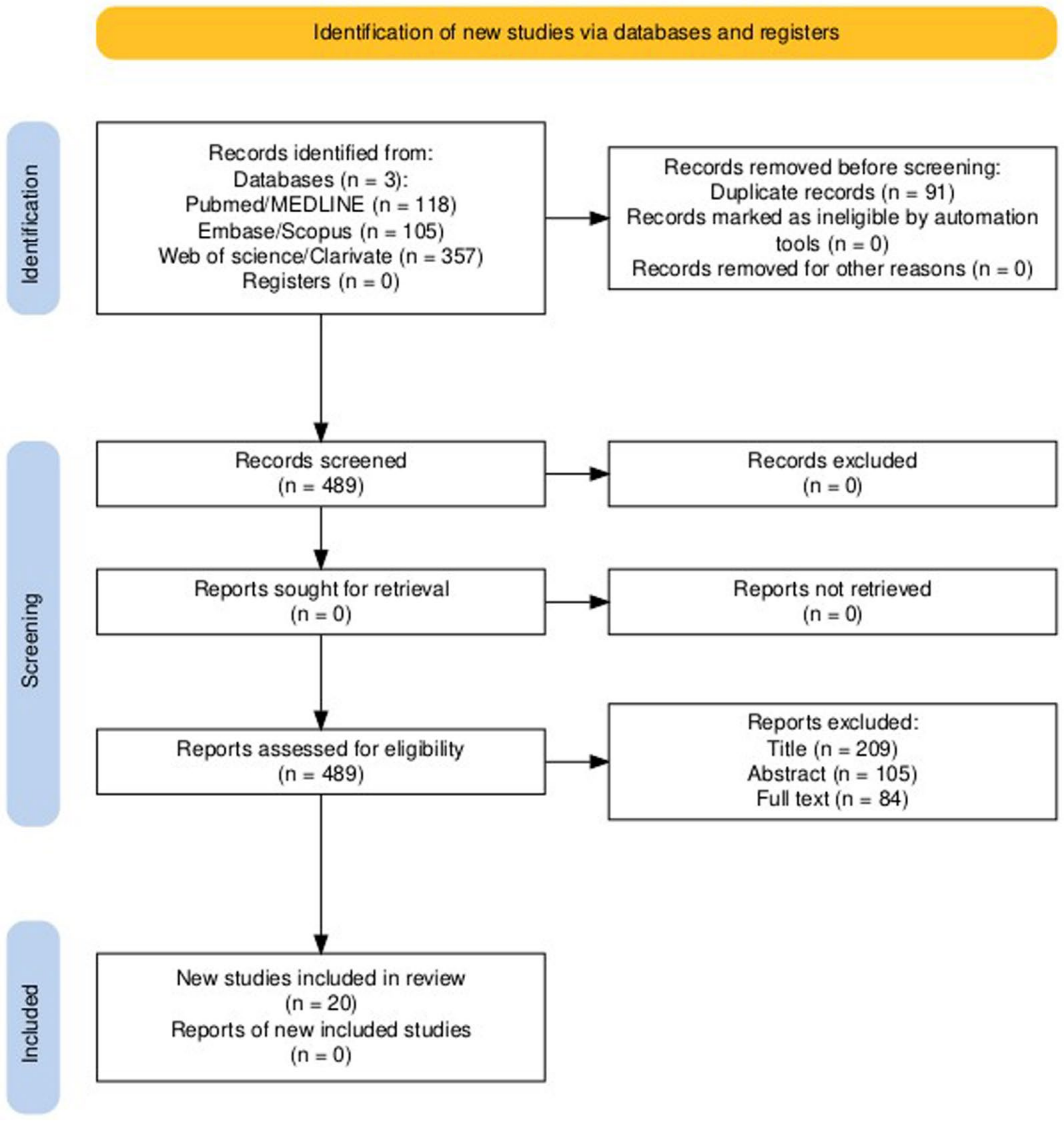
PRISMA flow diagram

In total, 209 were excluded by title, 105 were excluded by abstract, and 84 by full text, 20 were left for final analysis (Fig. 1) [33].

A notable mention among the excluded articles is Endo et *al*. [34], which was omitted from the final analysis owing to the lack of differentiation between retention of antibiotic spacers and revision to HA.

Owing to the heterogeneity of the studies, the different types of final implants, and the various scales used by the authors for evaluating shoulder function and pain at the last follow-up, it was not possible to perform a meta-analysis. Therefore, a descriptive approach and critical analysis of the results were conducted instead.

The final cohort that underwent conversion or revision to HA was comprised 268 patients: 91 (34%) of this cohort initially had a rTSA, 123 (46%) had an anatomic implant, 46 (17%) had a HA. Eight patients (3%), belonging to Grubhofer’s cohort had a not specified implant, either rTSA, aTSA or HA. Additionally, one patient (0.37%) from Streubel et al.’s study underwent conversion from humeral head resurfacing arthroplasty (HHR) to HA.

Revision to Shoulder HA was performed in case of failure of one or more components of aTSA or rTSA, HA, and HHR.

Glenoid component loosening was the predominant indication for conversion to HA, comprising the vast majority of aTSA → HA cases (110/124, ≈89%) and a clear majority of rTSA → HA cases (65/90, ≈72%), thus representing the largest indication overall. Soft-tissue–related problems were the next most frequent, particularly after prior HA → HA (soft-tissue failure 24/45, 53%), and to a lesser extent after aTSA → HA (6/124, ≈5%). The third most common indication was stiffness within the HA → HA conversion cohort (19/45; 42%). Infection was less common within the aTSA → HA and rTSA → HA cohorts (9/124 and 7/90, both ≈7%); eight additional conversions with unspecified index implant were reported in a two-stage setting (NS). Other indications included humeral loosening (10/124 after aTSA), instability (15/90 after rTSA; 2/45 after HA), and periprosthetic fractures (8/90 after rTSA) (Table 2).

After the first revision surgery, 202 patients received a revision HA, while 66 patients underwent a revision HA combined with glenoid grafting.

Complications were reported in 29% of total cases (Table 3) and 15% of the patients underwent further rerevisions as documented in seven papers, the remaining authors did not report reinterventions (Table 1).

**Table 3:** Complication spectrum after revision to HA

Complication	No. cases (%)
Not specified (NS)	31 (40)
Stiffness/pain	15 (19)
Painful glenoid erosion	10 (13)
Antero superior escape	05 (06)
Periprosthetic humeral fracture	04 (05)
Tuberosity lysis/migration	03 (04)
Rotator cuff tears	03 (04)
Humeral loosening	02 (02)
Humeral stress shielding	01 (01)
Clavicle fracture	01 (01)
Periprosthetic instability	01 (01)
Hematoma	01 (01)
Iliac crest fracture	01 (01)

**Fig. 2:**
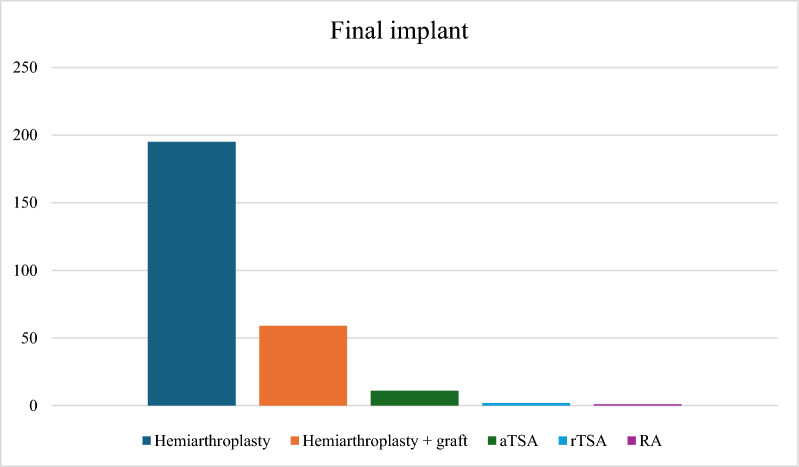
Graphic showing implant at the last follow-up. *aTSA* anatomic total shoulder arthroplasty; *rTSA * reverse total shoulder arthroplasty; *RA* resection arthroplasty

Following these reinterventions, revision HA alone was selected as the final implant in 195 cases; in 59 cases, glenoid grafting was carried out in conjunction with HA implantation. In 11 further cases the glenoid component was reimplanted and in two cases the HA was converted back to rTSA, conversely only one case was revised to a RA (Fig. 2).

## Clinical results

The average minimum follow-up was 12 months, and the mean follow-up was 58, 6 months.

Two studies encompassed in this review did not specify the functional evaluation scale [19, 29], and among the remaining studies only 12 authors presented the functional results after conversion/revision to HA.

ASES was the most frequently reported outcome measure, followed by the Constant score (Tables 4 and 5). SANE and SST were also commonly used as secondary measures, whereas SSV appeared less often (Table 5). Other instruments—including UCLA, QuickDASH, SPADI, University of Pennsylvania, and Modified Neer—were reported sporadically; their results are summarized in the following subsections (Tables 4 and 6).

**Table 4:** Functional outcomes: aTSA → HA

Indication	Author	Scoring system	No. patients	Preoperative average score	Postoperative average score
Glenoid loosening					
	Khoo	ASES	26	NR	65
	Nezwek	ASES	4	15	52
	Namdari	ASES	17	NR	58
	Khoo	Sane	26	NR	66
	Nezwek	Sane	4	25	70
	Namdari	Sane	17	NR	54
	Khoo	SST	26	4.4	7.4
	Nezwek	SST	4	3.3	8
	Sajadi	UCLA	7	10.1	17.1
Humeral loosening					
	Khoo	ASES	8	NR	66
	Khoo	Sane	8	NR	70
	Khoo	SST	8	3.7	8.5
	Sajadi	UCLA	2	12.5	17.5
Infection					
	Seitz	University of Pennsylvania score	5	NR	63
Instability					
	Khoo	ASES	6	NR	61
	Khoo	Sane	6	NR	57
	Khoo	SST	6	4	5.6

Functional results of conversion from aTSA to Hemi

**Table 5:** Functional outcomes: rTSA → HA

Indication	Author	Scoring system	No. patients	Preoperative average score	Postoperative average score
Glenoid loosening					
	Gamradt	ASES	3	NR	49.7
	Song	ASES	3	22.6	59.7
	Nezwek	ASES	4	15	52
	Glanzmann	Constant	8	24	25.2
	Kriechling	Constant	8	30	33
	Ladermann	Constant	28	23.7	37
	Nezwek	SST	4	8	3.3
	Gamradt	SST	3	NR	2.7
	Glanzmann	Q DASH	8	63.2	63
	Nezwek	SANE	4	25	70
	Glanzmann	SPADI	8	29.3	36.7
	Kriechling	SSV	8	28	35
	Song	UCLA	3	12.67	19
Instability					
	Nezwek	ASES	3	15	52
	Gamradt	ASES	1	NR	50
	Kriechling	Constant	2	30	33
	Nezwek	SANE	3	25	70
	Nezwek	SST	3	8	3.3
	Gamradt	SST	1	NR	1
	Kriechling	SSV	2	28	35
Infection					
	Glanzmann	Constant	3	24	25.2
	Glanzmann	Q DASH	3	63.2	63
	Glanzmann	SPADI	3	29.3	36.7
	Nezwek	ASES	3	15	52
	Gamradt	ASES	1	NR	50
	Nezwek	SANE	3	25	70
	Nezwek	SST	3	8	3.3
	Gamradt	SST	1	NR	1
Periprostetic	Scapular fracture				
	Glanzmann	Constant	4	24	25.2
	Kriechling	Constant	2	30	33
	Glanzmann	Q DASH	4	63.2	63
	Glanzmann	SPADI	4	29.3	36.7
	Kriechling	SSV	2	28	35
Periprostetic	Humeral fracture				
	Glanzmann	Constant	2	24	25.2
	Glanzmann	Q DASH	2	63.2	63
	Glanzmann	SPADI	2	29.3	36.7

Functional results of conversion from rTSA to Hemi

**Table 6:** Functional outcomes: HA → HA

Indication	Author	Scoring system	No. patients	Preoperative average score	Postoperative average score
Instability					
	Sajadi	UCLA	2	8.5	12.5
Soft tissue	Failure				
	Sajadi	UCLA	4	8.5	12.5

Functional results of revisions from Hemi to Hemi

### Revision from HA to HA

One study (Streubel) reported a single HHR to HA conversion assessed with the Modified Neer score; preoperative values were not provided and the postoperative outcome was unsatisfactory [35].

In standard HA revisions without implant exchange, Sajadi et al. reported similar UCLA improvements for both instability (*N* = 2: 8.5 → 12.5; Δ + 4.0) and soft-tissue failure (*N* = 4: 8.5 → 12.5; Δ + 4.0) (Table 6) [2]. Grubhofer et al. presented two-stage infection revisions to HA from non-specified index implants (NS, *N* = 8) with low postoperative scores (Constant 22; SSV 28) [26]. In the largest HA → HA series (Gauci et al.), functional scores were not reported, precluding comparison [19].

### Revision from aTSA to HA

Across the seven studies reporting conversions from aTSA to HA, outcomes varied by indication (Table 4). Khoo observed the highest postoperative scores after humeral loosening (ASES 66; SANE 70; *N* = 8) and similarly favorable results in glenoid loosening (ASES 65; SANE 66; SST 7.4; *N* = 18), whereas soft-tissue failure yielded lower values (SANE 57; SST 5.6) [27]. Namdari reported inferior means in glenoid loosening (ASES 58; SANE 54; *N* = 17), plausibly reflecting larger glenoid vault defects requiring bone grafting [24]; Aibinder provided Modified Neer scores for glenoid loosening at 8.3-year follow-up (*N* = 11) but without preoperative values, limiting interpretability [36]. Infection-related revisions showed the poorest function: Grubhofer reported Constant 22 and SSV 28 in a staged setting (not specified index implants) [26], whereas Seitz (aTSA → spacer → HA, *N* = 5) reported a University of Pennsylvania score of 63 after cancellous grafting with biologic resurfacing, suggesting that glenoid and soft-tissue management can modulate recovery [3]. Preoperative data were inconsistently available across studies, so Δ could not be calculated in several cohorts, and subgroup denominators—particularly for humeral loosening—were small; these trends should therefore be interpreted with caution.

### Revision from rTSA to HA

Across rTSA to HA conversions, outcomes differed by indication (Table 5). Glenoid loosening generally yielded higher postoperative function than instability, with infection showing the poorest results. Song reported ASES means of 59.7 for glenoid loosening (*N* = 3) and 53.3 for instability (*N* = 3) [25]. Gamradt observed the lowest scores in instability/infection cohorts, whereas glenoid loosening reached ASES 49.7; SST was 2.7 for glenoid loosening and 1 for infection/instability [37]. In a severe bone-loss series, Nezwek reported pooled ASES 52 across indications (largest share glenoid loosening), with SANE 70 and SST 3.3 [38]. On Constant scoring, Lädermann (glenoid loosening) reported 37 (*N* = 28) [28], Kriechling reported 33 across mixed rTSA indications (12 patients) (SSV 35) [30], and Glanzmann reported 25.2 with additional disability metrics (QuickDASH 63, SPADI 36.7) [39]. Low postoperative values in infection-driven two-stage revisions were also described by Grubhofer (Constant 22, SSV 28), although those HA conversions derive from non-specified index implants (NS) rather than exclusively rTSA [26]. Preoperative values were inconsistently reported across studies, preventing calculation of Δ in several cohorts; small subgroup denominators further limit generalizability. Taken together, glenoid loosening outperforms instability, and infection yields the poorest results, with the largest subgroup (Lädermann, *N* = 28) driving the most stable Constant estimates.

### Complications and reinterventions

The authors reported a total of 77 complications accounting for 29% of total cases (Table 3): 62 were postoperative complications, 12 intraoperative, and 3 were not specified by the author.

These complications led to 42 reoperations (54.77% of all complications; 15.7% of the total implants): Sajadi et al. reported two glenoid reimplantations due to persistent painful glenoid [2], among Glanzmann’s cohort of patients, one patient, who experienced shoulder pain due to excessive glenoid medialization, was converted to resection arthroplasty [39].

Aibinder reported two intraoperative humeral fractures, treated with cerclage wiring; postoperatively, two patients experienced medial glenoid erosion. One underwent a simple reimplantation of the glenoid component, while the other underwent reimplantation of the glenoid component coupled with posterior capsule plication [36].

Grubhofer et al. reported five reinterventions without specifying which kind of complication or reintervention did the patient’s experience [26].

In Namdari’ study, five patients underwent further reintervention for glenoid component reimplant due to postoperative glenoid sided arthrosis [24].

In Gauci’s retrospective analysis 20 reinterventions were performed after revision to HA, the main causes were stiffness or pain in 12 cases, tuberosity lysis or migration in 3 cases, cuff tear in 3 cases, periprosthetic instability, and hematoma accounted for 1 case each; reinterventions were carried out without exchange of the implant [19].

Kriechling reported one intraoperative complication in the form of Iliac crest fracture, treated with plate fixation [30].

Overall, seven patients belonging to Khoo’s cohort required additional surgery: four underwent revision to total shoulder arthroplasty (two anatomic and two reverse), while three had revision HA to address pain and stiffness [27].

## Discussion

Stratifying by initial implant and indication clarifies where HA offers meaningful benefit. Across studies, mechanical loosening—particularly humeral loosening after aTSA—showed the highest postoperative scores [27], while glenoid loosening (after aTSA or rTSA) produced consistent, moderate improvements [25, 27, 38]. In contrast, infection yielded the poorest outcomes [26, 39]. These trends are reflected in Tables 4 and 6 and are concordant with the overall complication (29%) and reoperation (15.7%) rates (Table 3).

Evidence for HA-to-HA conversion is sparse and heterogeneous. The only HHR-to-HA case [35] reported an unsatisfactory Modified Neer outcome, with no preoperative value available. In standard HA revisions without implant exchange, Sajadi documented comparable UCLA improvements for both instability and soft-tissue failure [2], as summarized in Table 6. Notably, the largest HA → HA series by Gauci did not report functional scores, which prevents direct comparison with other cohorts [19].

Outcomes after conversion from aTSA were clearly indication-dependent (Table 4). Humeral loosening yielded the best postoperative results in Khoo’s series, although this subgroup represents only a small fraction (~3%) of the overall cohort, limiting generalizability [27]. Glenoid loosening performed consistently well in the same study, while soft-tissue failure scored lower [27], conversely Namdari et al. reported inferior means for glenoid loosening, plausibly reflecting larger glenoid vault defects requiring grafting [24]. Infection produced the poorest function [26], whereas Seitz et al. reported a University of Pennsylvania score of 63 after cancellous grafting plus biologic resurfacing, suggesting that glenoid reconstruction can improve postoperative recovery [3].

In the rTSA → HA cohort, glenoid grafting was common: Nezwek grafted 7/7 cases and added biologic resurfacing, Lädermann 11/28, Gamradt 4/4, and Kriechling 7/12, the total of patients was 29/51 (≈57%) across these series. Despite this unfavorable anatomy, functional outcomes were moderate and clinically meaningful in several reports [28, 30, 38]. By contrast, cohorts dominated by instability and/or infection [26, 37] showed low postoperative scores.

Taken together, glenoid loosening tends to outperform instability, and infection yields the poorest results. Part of the advantage in glenoid loosening likely reflects that bone loss can be reconstructed: where stable graft fixation is feasible, HA can deliver salvage-level improvements [28, 30, 38]. Adjunct measures may matter as well—Nezwek’s biologic resurfacing alongside grafting coincided with favorable patient-reported scores despite severe defects [38]. These comparisons should be interpreted cautiously: grafted cohorts differ systematically from nongrafted ones (selection bias), preoperative scores are inconsistently reported (limiting Δ), and radiographic endpoints (e.g., graft incorporation) are variably detailed.

A notable limitation of the literature is the lack of standardized functional outcome measures, which hampers direct comparisons across studies; this issue has also been emphasized in prior systematic reviews [40]. Our review is likewise limited by heterogeneity in revision indications and assessment tools, small subgroup sizes (restricting generalizability), and the absence of randomized controlled trials. Future work should prioritize standardized reporting (including pre- and postoperative values for each scale), larger indication-specific cohorts, and longer follow-up to refine indications and optimize patient outcomes.

## Conclusions

Hemiarthroplasty (HA) is a salvage option for revision shoulder arthroplasty when reimplantation (aTSA/rTSA) or secure glenoid fixation is not feasible. Its effectiveness is indication dependent. The best postoperative function is observed after humeral loosening (a small subgroup, limiting generalizability), while glenoid loosening typically achieves moderate, clinically meaningful improvements, particularly when bone loss can be reconstructed. By contrast, instability yields only modest gains, and infection is associated with the poorest outcomes and higher adverse-event rates.

Given these patterns and the overall complication and reoperation burden, HA should be selected deliberately, with indication-specific counseling and realistic expectations. Where undertaken, attention to glenoid bone management and soft-tissue status may enhance results.

Future work should prioritize standardized reporting (pre-/postoperative values and scale-specific denominators), prospective, indication-stratified cohorts, and targeted comparisons of HA against alternative salvage strategies to better define when HA offers the greatest value.

## Data Availability

All relevant data supporting the findings of this review, including extracted data and summary tables, are presented in the main manuscript and supplementary materials. As this study is based on a review of published literature, no raw datasets were generated for public deposition. The figures and tables used in this review are provided as part of the manuscript submission.

## Abbreviations

HA: Hemiarthroplasty
rTSA: Reverse Total Shoulder Arthroplasty
aTSA: Anatomic Total Shoulder Arthroplasty
RA: Resection Arthroplasty
PRISMA: Preferred Reporting Items for Systematic Reviews and Meta-Analyses
ROM: Range of Motion
CA: Coracoacromial
ASES: American Shoulder and Elbow Surgeons (score)
SANE: Single Assessment Numeric Evaluation (score)
SST: Simple Shoulder Test (score)
SPADI: Shoulder Pain and Disability Index (score)
SSV: Subjective Shoulder Value (score)
UCLA: University of California, Los Angeles (score)
PSI: Patient-Specific Implants
CT: Computed Tomography
HHR: Humeral Head Resurfacing
RCT: Randomized Controlled Trials
